# Superresolution Imaging Identifies That Conventional Trafficking Pathways Are Not Essential for Endoplasmic Reticulum to Outer Mitochondrial Membrane Protein Transport

**DOI:** 10.1038/s41598-017-00039-5

**Published:** 2017-02-02

**Authors:** Kyle Salka, Shivaprasad Bhuvanendran, Kassandra Wilson, Petros Bozidis, Mansi Mehta, Kristin Rainey, Hiromi Sesaki, George H. Patterson, Jyoti K. Jaiswal, Anamaris M. Colberg-Poley

**Affiliations:** 1 Center for Genetic Medicine Research, Children’s National Health System, 111 Michigan Ave, NW, Washington, DC 20010 USA; 2 Laboratory of Microbiology, Department of Medicine, School of Health Sciences, University of Ioannina, Ioannina, 45500 Greece; 3 Section on Biophotonics, National Institute of Biomedical Imaging and Bioengineering, National Institutes of Health, Bethesda, MD 20892 USA; 4 Department of Cell Biology, Johns Hopkins University School of Medicine Hunterian 111, 725 N. Wolfe Street, Baltimore, MD 21205 USA; 5 Department of Integrative Systems Biology, and of Pediatrics, George Washington University School of Medicine and Health Sciences, Washington, DC 20037 USA; 6 Departments of Biochemistry & Molecular Medicine, and of Microbiology, Immunology & Tropical Medicine, George Washington University School of Medicine and Health Sciences, Washington, DC 20037 USA

## Abstract

Most nuclear-encoded mitochondrial proteins traffic from the cytosol to mitochondria. Some of these proteins localize at mitochondria-associated membranes (MAM), where mitochondria are closely apposed with the endoplasmic reticulum (ER). We have previously shown that the human cytomegalovirus signal-anchored protein known as viral mitochondria-localized inhibitor of apoptosis (vMIA) traffics from the ER to mitochondria and clusters at the outer mitochondrial membrane (OMM). Here, we have examined the host pathways by which vMIA traffics from the ER to mitochondria and clusters at the OMM. By disruption of phosphofurin acidic cluster sorting protein 2 (PACS-2), mitofusins (Mfn1/2), and dynamin related protein 1 (Drp1), we find these conventional pathways for ER to the mitochondria trafficking are dispensable for vMIA trafficking to OMM. Instead, mutations in vMIA that change its hydrophobicity alter its trafficking to mitochondria. Superresolution imaging showed that PACS-2- and Mfn-mediated membrane apposition or hydrophobic interactions alter vMIA’s ability to organize in nanoscale clusters at the OMM. This shows that signal-anchored MAM proteins can make use of hydrophobic interactions independently of conventional ER-mitochondria pathways to traffic from the ER to mitochondria. Further, vMIA hydrophobic interactions and ER-mitochondria contacts facilitate proper organization of vMIA on the OMM.

## Introduction

Mitochondria consist of nearly a thousand proteins, and aside from the 13 proteins encoded by the mitochondrial genome, the rest are encoded by nuclear genes^1^. These proteins are synthesized in the cytosol and imported into mitochondria using highly conserved translocation machinery^2^. Analysis of the mitochondrial proteome has identified that a number of these proteins also localize in other organelles including over fifty proteins that are classified as endoplasmic reticulum (ER) proteins^3^. Cellular proteins that traffic to mitochondria via the ER include apoptosis inducing factor (AIF), acyl-CoA:diacylglycerol acyl-transferase 2 (DGAT2), and retinol dehydrogenase 10 (Rdh10), which traffic directly from the ER to the mitochondria^4–6^. Aside from cellular proteins, pathogen-encoded proteins such as the human cytomegalovirus (CMV) encoded viral mitochondrial-localized inhibitor of apoptosis (vMIA), hepatitis c virus (HCV) encoded N3/4A protease, and human immunodeficiency virus 1 (HIV-1) encoded viral protein R (Vpr) also traffic from the ER to mitochondria^7–12^.

There are two routes proposed for protein trafficking from the ER to mitochondria. The first is based upon ER and the OMM proximity, where a bridge (tether) facilitates calcium (Ca^2+^) transfer through the mitochondria-associated membrane (MAM) calcium signaling complex, which contains inositol 1,4,5 trisphosphate receptors (IP3Rs), cytosolic glucose response protein 75 (Grp75) and the outer mitochondrial membrane (OMM)-localized voltage dependent anion channel (VDAC), and lipids between these compartments^13–16^. In yeast, MAM tethers, known as ER mitochondria encounter structure (ERMES) facilitate phospholipid exchange^17^. ER-OMM contacts may facilitate transfer of proteins between these compartments. In mammalian cells, several proteins including phosphofurin acidic cluster sorting protein 2 (PACS-2), Nogo (or reticulon 4) and mitofusins (Mfn1/2) have been implicated in regulating ER-mitochondrial apposition^14,18–21^. PACS-2 is required for proper distribution of the MAM-enriched protein calnexin^22^. It is currently debated whether mitofusins regulate ER-mitochondrial tethering and mitochondrial Ca^2+^ uptake in positive or negative manner. Although homotypic interactions between Mfn2 and heterotypic interaction with Mfn1 have been implicated in decreasing ER-mitochondria tethering and functional coupling^23,24^, a recent study re-established the previous report that Mfn2 is an ER-mitochondrial tether and its ablation reduces mitochondrial Ca^2+^ uptake without altering the mitochondrial Ca^2+^ uniporter complex^21,25^. While the precise mechanism of action of mitofusins in ER-mitochondria coupling is yet to be resolved, lack of Mfn1/2 has been shown to affect the distribution of proteins at the OMM by altered MAM tethering^26^. The second route for protein trafficking at the MAM involves vesicular transport from ER to mitochondria, where membrane scission protein called dynamin related protein 1 (Drp1) facilitates transport of proteins from the ER to mitochondria^4,10^. Subpopulations of AIF and HIV-1 viral protein Vpr are packaged and transported to mitochondria in vesicles. Knockdown of Drp1, ATPase family AAA domain containing 3A (ATAD3A), or Mfn2 decreases AIF and HIV Vpr trafficking to mitochondria^4,10^. Drp1, ATAD3A and Mfn2 are suggested to play distinct roles by facilitating budding, movement and fusion of the vesicles, respectively.

Similar to the mitochondrial signal-anchored proteins, which traffic from the cytosol to the OMM, we found that CMV vMIA is signal-anchored by an N-terminal single pass hydrophobic leader that serves as part of its mitochondrial targeting signal (MTS)^7,11^. Rather than direct transport from the cytosol to the OMM, we previously showed that vMIA traffics sequentially from the ER to mitochondria through MAM contacts between the two organelles^7–9,11^. vMIA signal sequence is not cleaved through its trafficking to OMM, identifying that its transport does not involve transiting through the Golgi complex. However, the role of host proteins in vMIA trafficking has not yet been studied. Notably, vMIA targeting is efficient: vMIA is able to retarget a cellular ER protein, viperin, to mitochondria and the vMIA MTS can retarget the Tom20 hydrophobic leader to this ER to mitochondrial trafficking^11,27^. Thus, vMIA offers a valuable tool to define the mechanism for ER to mitochondrial trafficking of signal-anchored OMM proteins.

While no consensus MAM/mitochondrial targeting signal is known, the factors affecting trafficking at the MAM are now emerging^11,22^. Affinity for lipids has been implicated in MAM trafficking of proteins including the lipid synthetic proteins. Targeting of Rdh10 to the MAM and mitochondria requires its N- and C-terminal hydrophobic domains and Rdh10 can relocalize to lipid droplets^6^. Similarly, cellular retinol-binding protein type 1 localizes to the MAM and mitochondria and can relocalize to lipid droplets^6,28^. Palmitoylation enriches calnexin and thioredoxin related transmembrane protein in the MAM^29^. Sigma 1 receptor (Sig-1R) localizes to the MAM using cholesterol binding^30,31^. Similarly, the N-terminal MTS of vMIA contains an evolutionarily conserved hydrophobic leader, with a consensus cholesterol binding domain (CBD) and multiple basic residues and proline rich domain (PRD) downstream of the CBD^7,11^. We found that vMIA mutations that increase its MTS hydropathy score retarget vMIA to the secretory apparatus and reduce its mitochondrial trafficking^11^. Mutations in the vMIA CBD block its association with MAM lipid rafts, but do not affect its trafficking to the OMM^32^.

Similar to many other OMM proteins, including Tom20, Tom22, VDAC, and the associated cytosolic hexokinase I^33–35^, vMIA also organizes in nanoscale clusters^13,36^. Using superresolution imaging by multifocal structured illumination microscopy (MSIM), gated stimulated emission depletion (gSTED) and photoactivated localization microscopy (PALM), we established that vMIA forms clusters of ~100–150 nm at the OMM of human cells^13,36^. Clustering has also been reported for inner mitochondrial membrane (IMM) proteins including mitochondrial inner membrane or cristae organizing system (MICOS or MINOS), and cytochrome C oxidase subunit 2 of complex IV^35,37^. These mitochondrial protein clusters are not static as they can change in response to the functional requirements of mitochondria^34^, and following viral infection^38^. However, the cellular mechanism for facilitating clustering of mitochondrial proteins and how this is dynamically regulated is poorly understood.

In this study, we investigated the cellular mechanisms reported for ER to OMM protein trafficking and OMM clustering of a membrane-anchored protein. To test the requirements of ER-mitochondria apposition and tethering (bridge model) for trafficking, we used PACS-2 null and Mfn1/2 null mouse embryonic fibroblasts (MEFs)^39,40^ and PACS-2 knockdown human cells. To investigate the role of membrane scission (vesicle model) in trafficking from ER to mitochondria, we used Drp1-null cells^41^. Additionally, by using vMIA MTS mutants, we have assessed the role of hydrophobic and lipid interactions on mitochondrial localization of vMIA and its ability to dimerize and form clusters at the OMM. Our results identify that vMIA uses an unconventional hydrophobic interaction-mediated mechanism to traffic from the ER to the OMM and does not require Drp1-, PACS-2-, and mitofusin-mediated pathways. Instead, PACS-2 and Mfn-mediated ER-mitochondria apposition regulates the ability of vMIA to form clusters at the OMM.

## Results

### vMIA traffics to and clusters at the OMM in MEFs

**Figure 1:**
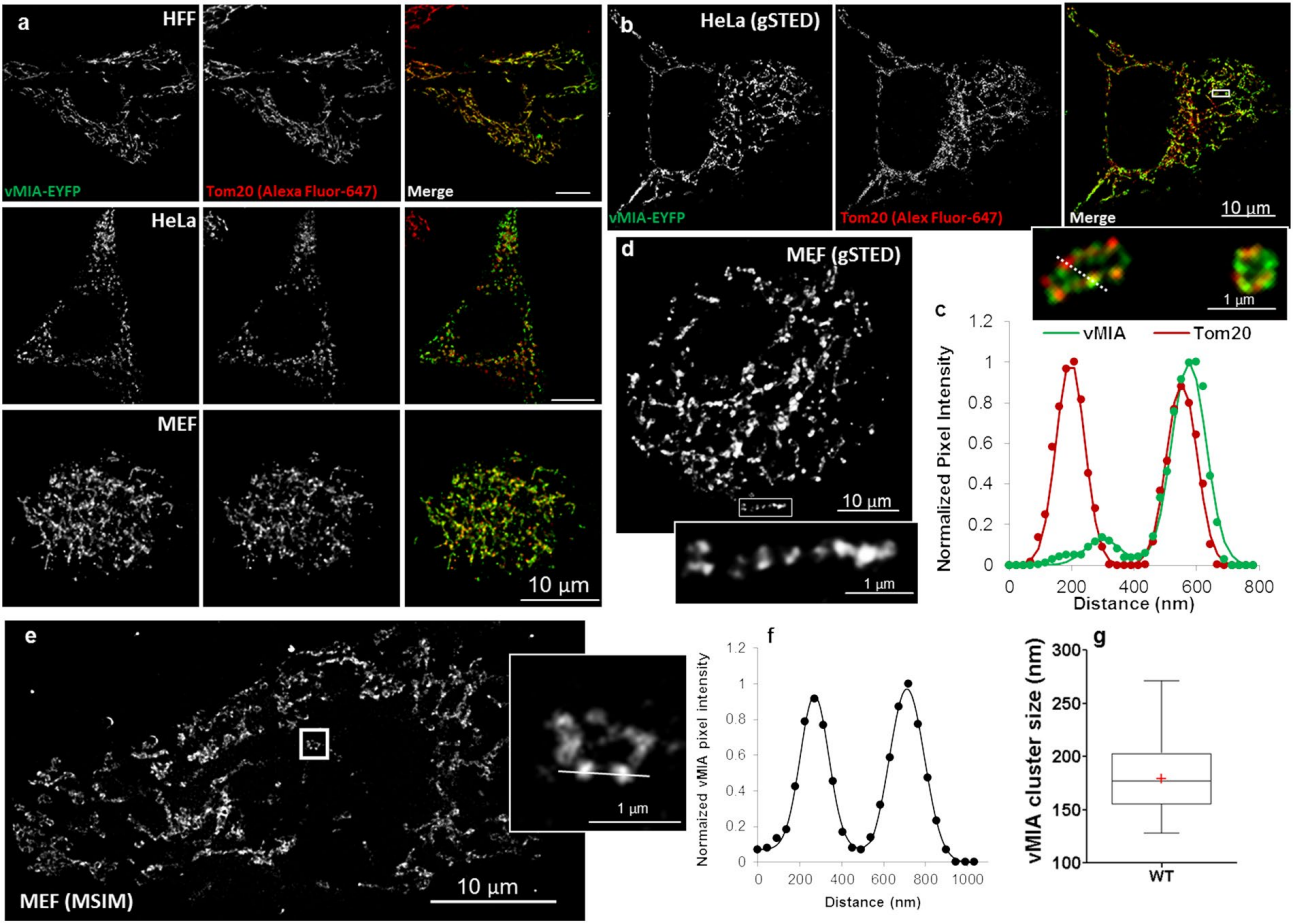
vMIA traffics to and clusters at the OMM in mouse embryonic fibroblasts. (**a**) Human (HFF and HeLa) and mouse (MEF) fibroblasts were transfected transiently (19 hours) to express vMIA-EYFP and then immunolabeled for the OMM marker Tom20. The images show an optical slice obtained by deconvolution of a confocal z-stack. (**b**) A single optical plane from deconvolved z-stack of gSTED images showing clustered distribution of vMIA-EYFP and Tom20 (immunostained) on the OMM of a HeLa cell transfected as above. Zoom shows the merged image marked by white box in the merged channel. (**c**) Normalized intensity profile of the red and green pixels marked by the dotted line in the zoomed region in panel b. (**d**) Single optical plane from deconvolved z-stack of gSTED images showing clustered distribution of vMIA-EYFP on the OMM in a WT MEF. Zoom shows the region marked by white box. (**e**) A single plane from an MSIM z-stack images showing clustered distribution of vMIA-EGFP on the OMM of a transfected MEF cell. Zoom of a single mitochondrion (boxed) shows vMIA clustering and line marks the region used to plot the intensity profile in the panel d. (**f**) Normalized intensity profile of the mitochondrion along the pixels marked by the solid line in the zoomed region. (**g**) Plot showing vMIA cluster size (FWHM) in WT MEFs. The line in the box and red, cross mark indicate the median (177.0) and mean (179.9 ± 3.7), respectively (n = 75).

vMIA traffics from the ER to OMM in human cells including human primary fibroblasts (HFFs), U373 and HeLa cells^7–9,11,13,36^. By using confocal microscopy and gSTED superresolution microscopy of vMIA with the OMM marker Tom20, we investigated the localization of vMIA at the OMM in human and mouse cells. Confocal microscopy confirmed mitochondrial localization of vMIA in all (human and mouse) cells tested (Fig. 1a). Pixel co-localization analysis in WT-MEFs identified 83.9 ± 0.5% co-localization between vMIA and Tom20 (Fig. S1), showing that, similar to the human primary fibroblasts, vMIA traffics efficiently to the OMM in WT MEFs.

Using gSTED superresolution imaging, we examined if the vMIA clusters that we previously described in the human cells^13,36^ localize with Tom20 clusters. Using HeLa cells, we found that vMIA exists in clusters that can include or exclude Tom20 clusters (Fig. 1b inset, and corresponding line intensity profile in 1c). Use of gSTED imaging together with MSIM superresolution imaging of the wild type (WT) MEFs showed that vMIA also formed clusters at the OMM in mouse cells (Fig. 1d–g). MSIM analysis of vMIA clustering at the OMM in MEFs identified that the full width half maximum (FWHM) of vMIA clusters is 179.9 ± 3.7 nm (n = 75) (Fig. 1c,d). This size is similar to 100–150 nm sized clusters we previously detected in the human cells^13,36^. Together, above results identify that vMIA mitochondrial trafficking and organization in clusters at the OMM occurs similarly in human and mouse cells.

### vMIA forms homodimers in MEFs

**Figure 2:**
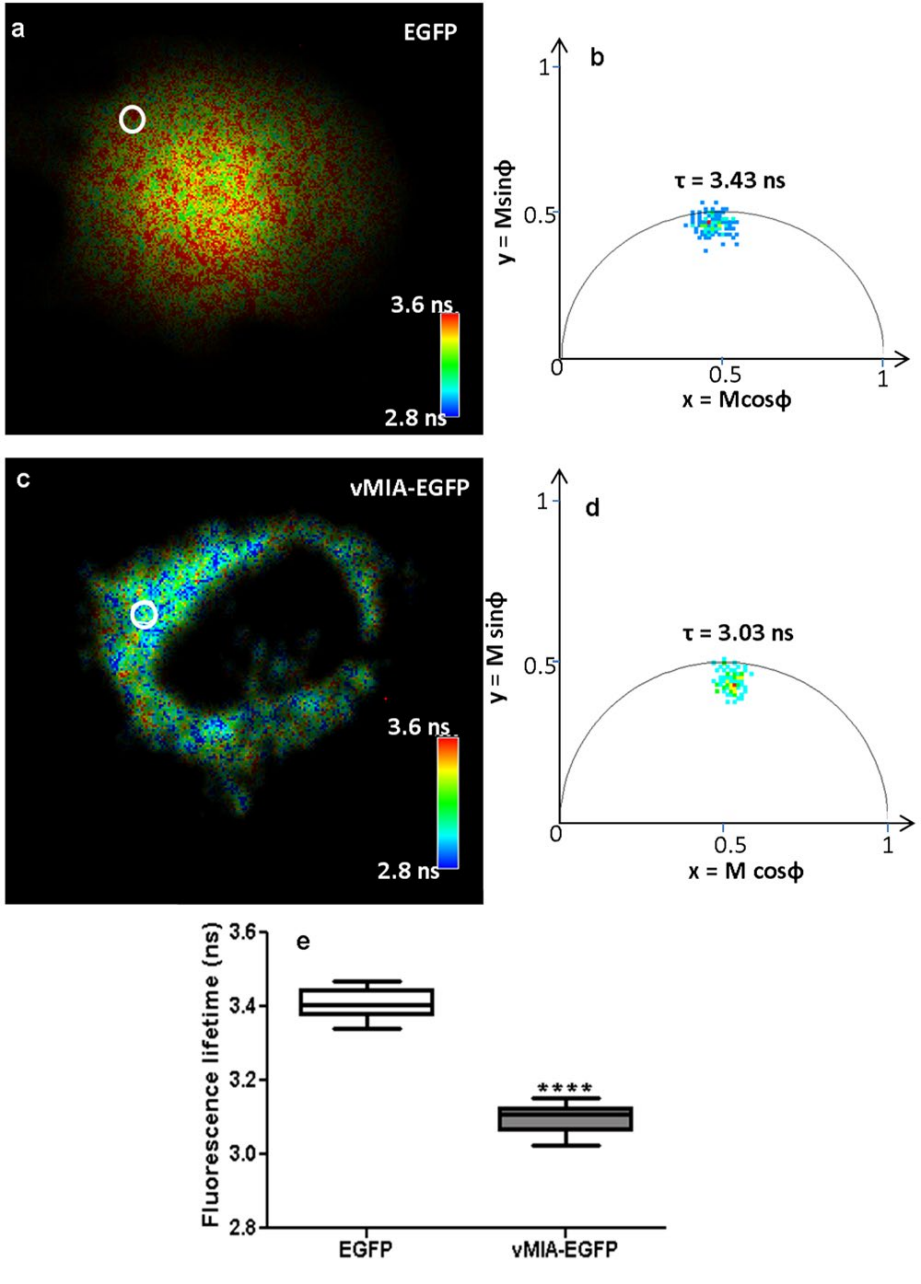
Use of fluorescence lifetime imaging to monitor vMIA homodimerization in WT MEFs. vMIA-EGFP was transiently expressed (as above) in WT MEFs, and live cells were imaged by using frequency domain lifetime microscopy. (**a**) Fluorescent lifetime image of a WT MEF expressing cytosolic EGFP. The pixels are pseudocolored based on their lifetime values as indicated by the heat map scale. (**b**) Fluorescence lifetime polar plot representing the frequency characteristics of each pixel in the ROI marked by the white circle in panel a. The colored squares represent a unique pixel lifetime and the red square marks the median lifetime value of the ROI (**c**) Fluorescent lifetime image of a WT MEF expressing vMIA-EGFP. The pixels are pseudocolored based on their lifetime as in panel b. (**d**) Polar plot presentation of the fluorescence lifetime of the ROI marked by white circle in panel c. The plot coordinates for (**b**) and (**d**) are x = M cosϕ and y = M sinϕ, where M is the modulation and ϕ is the phase delay. (**e**) The fluorescence lifetime comparison of cytosolic EGFP (τ = 3.41 ± 0.01 ns, n = 140 ROIs from 14 cells) and vMIA-EGFP (τ = 3.10 ± 0.01 ns, n = 130 ROIs from 13 cells) in WT MEFs. ****Represents p < 0.0001.

vMIA is known to homodimerize during ER to mitochondrial trafficking in human (HFFs and HeLa) cells^42,43^. To monitor this process in mouse cells, we imaged live cells using fluorescent lifetime imaging microscopy (FLIM) (Fig. 2). As has been demonstrated before, homoFRET imaging allows monitoring protein-protein proximity by change in fluorescence lifetime^44^. We thus imaged EGFP lifetime in WT MEFs expressing cytosolic EGFP or vMIA-EGFP. This shows a homogenously greater pixel lifetimes in cell expressing EGFP (Fig. 2a,b), but a more heterogeneous pixel lifetime values in a cell expressing vMIA-EGFP (Fig. 2c,d). Quantification of lifetime of the pixels (e.g.: those marked by the circles in Fig. 2a,c) showed the average lifetime of cytosolic EGFP was 3.41 ± 0.01 ns (10 regions per cell, n = 14 cells), which was reduced to 3.10 ± 0.01 ns for vMIA-EGFP (10 regions per cell, n = 13 cells, p < 0.0001) (Fig. 2e). This decrease in EGFP lifetime when tagged to vMIA demonstrates that, similar to human cells, vMIA efficiently homodimerizes during its trafficking from ER to mitochondria in MEFs. The quantitative nature of FLIM also provides an assay to quantify alterations in vMIA homodimerization on a single cell basis. We have used this ability in subsequent sections to study the effects of various genetic changes in host cells and in vMIA itself on its ability to form homodimers.

### Mfn2 affects vMIA clustering at the OMM

**Figure 3:**
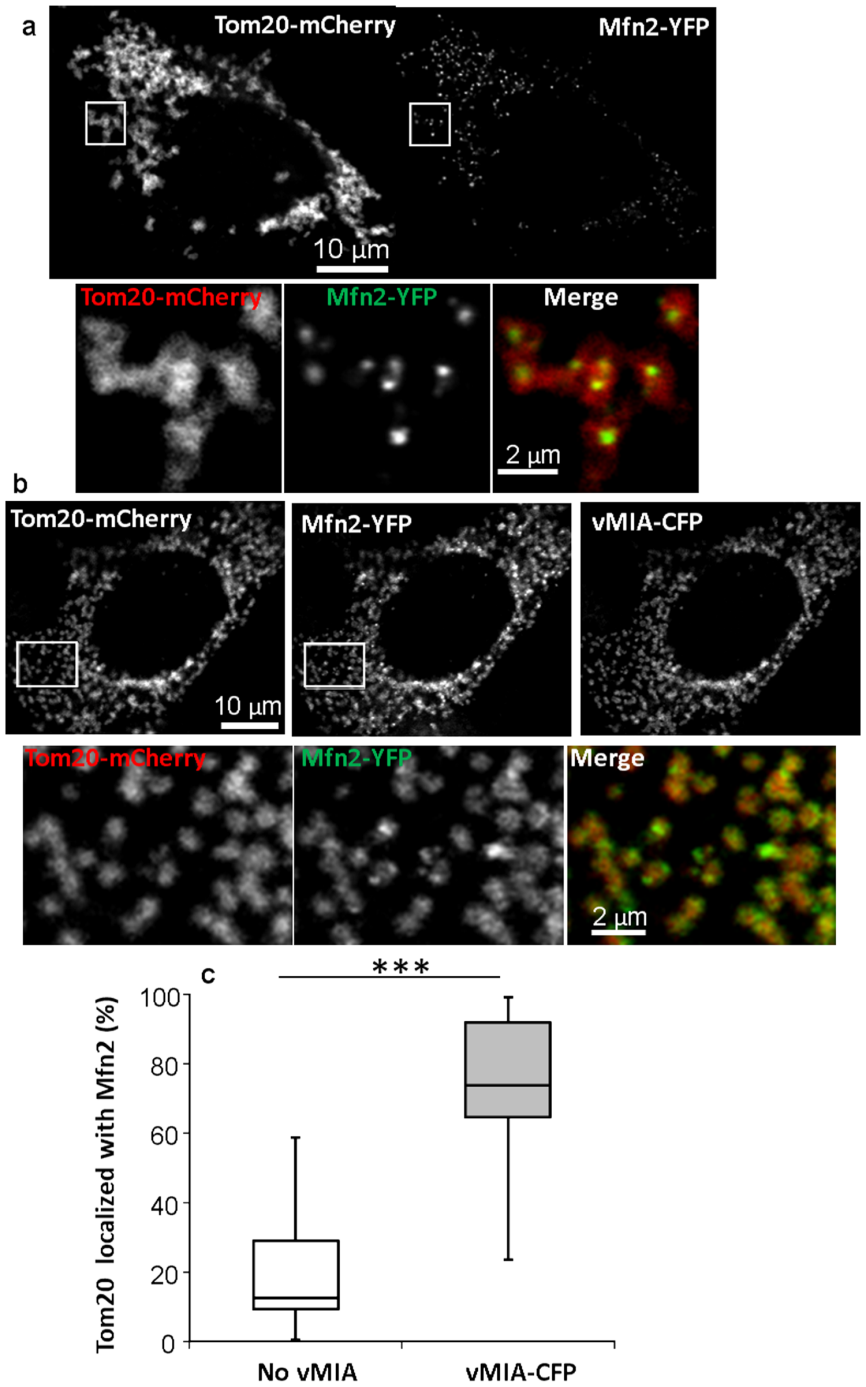
Expression of vMIA causes redistribution of Mfn2 on the OMM. MEFs transfected with Mfn2-YFP (pseudocolored green) and Tom20-mCherry (red), (**a**) without or (**b**) with vMIA-CFP (blue) were analyzed by confocal microscopy. (**a**) In MEFs not expressing vMIA, Mfn2 shows punctate distribution along the mitochondria; boxes mark the zoomed regions shown below. (**b**) Expression of vMIA causes Mfn2 to distribute more uniformly along the mitochondria; boxes mark the zoomed regions below. (**c**) Plot showing quantification of Mfn2 colocalization with OMM marker Tom20 in MEFs expressing or not expressing vMIA-CFP. The line in the box represents the median value of cells not expressing (13%) or expressing vMIA (73.9%) (n > 16 cells). ***Represents p < 0.001.

Mfn1/Mfn2 are proposed to tether the ER to mitochondria^21,25^, and interaction of vMIA with Mfn2 is important for its anti-apoptotic function^45^. Thus, we hypothesized that mitofusin-vMIA interactions may regulate vMIA trafficking from ER to OMM. To test this, we first examined vMIA and Mfn2 colocalization in MEFs (Fig. 3). In MEFs, Mfn2 localizes at mitochondrial junctions, causing Mfn2-YFP (green) to localize with the OMM marker, Tom20-mCherry (red) (89 ± 3.4%) (Fig. 3a). However, unlike Tom20, Mfn2 forms discreet puncta at the mitochondrial junction sites, causing only a small percentage of Tom20 pixels (18 ± 4.1%) at the OMM to overlap with the Mfn2 pixels (Fig. 3c). Expression of vMIA in MEFs caused redistribution of Mfn2 along the mitochondria (Fig. 3b). While the punctate distribution of Mfn2 was still detectable, Mfn2 redistributed along the entire OMM such that colocalization of Tom20 pixels with Mfn2 increased from 18 ± 4.1% to 75 ± 5.1% (Fig. 3b,c). Mfn2 also colocalized well with vMIA (85 ± 3.4%; Fig. 3b).

**Figure 4:**
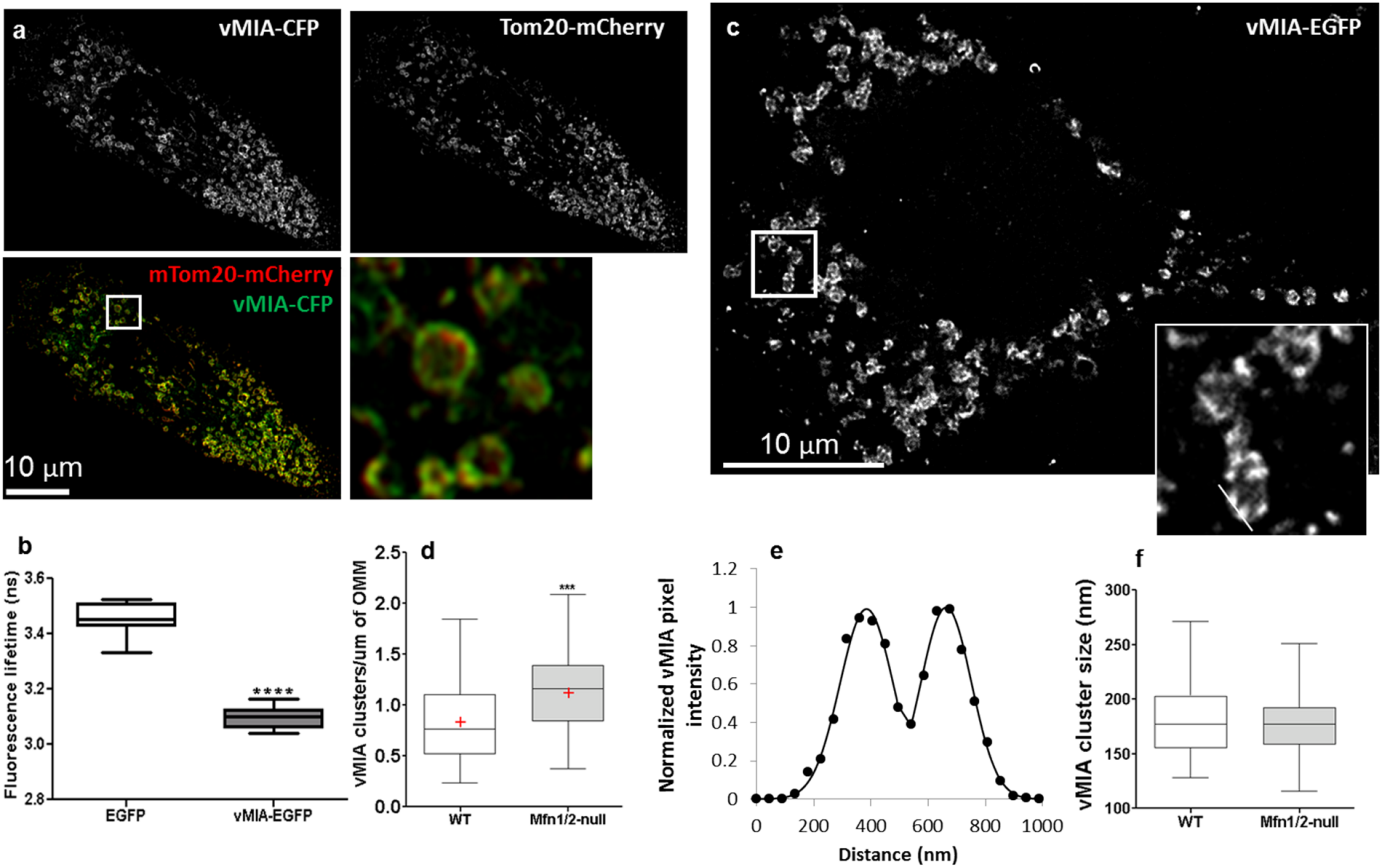
Role of mitofusins (Mfn1/2) on vMIA trafficking and clustering on mitochondria. (**a**) Mfn1/2-null MEFs were transiently transfected to express vMIA-CFP (pseudocolored green) and Tom20-mCherry (red) and imaged by confocal microscopy. The images show a single confocal plane from a deconvolved z-stack presented as monochrome images of the individual channels, which are pseudocolored for the merged image, and the boxed region marks the region zoomed in the inset. (**b**) The fluorescence lifetime comparison of cytosolic EGFP (τ = 3.46 ± 0.015 ns, n = 150 regions from 15 cells) and vMIA-EGFP (τ = 3.10 ± 0.011 ns, n = 130 regions from 13 cells) in Mfn1/2-null MEFs. (**c**) A single plane from an MSIM z-stack images showing clustered distribution of vMIA-EGFP on the OMM of an Mfn1/2-null MEF. A zoom of mitochondria shows vMIA clustering. (**d**) The number of vMIA clusters/µm of the OMM in WT MEFs (n = 50) and Mfn1/2-null MEFs (n = 40) are shown. (**e**) Normalized intensity profile along the solid line shown in the zoomed inset in panel c. (**f**) The FWHM of vMIA clusters (n = 40) in Mfn1/2-null MEFs were measured and plotted. The line and red cross mark indicate the median and mean, respectively. ****Represents p < 0.0001.

In view of the redistribution of Mfn2 upon vMIA expression, we investigated whether mitofusins affect vMIA trafficking. The two mitofusin proteins (Mfn1 and Mfn2) have been shown to be partly redundant, as overexpression of either protein is able to rescue the loss of the other^46^. In view of this partial redundancy in the function of the mitofusins, to test if Mfn2 is required for vMIA trafficking from the ER to OMM, we made use of Mfn1/2-null MEFs (Fig. 4). Similar to the extensive colocalization of vMIA-CFP with Tom20-mCherry in WT MEFs (83.9 ± 0.5%; Fig. S1), vMIA colocalized with Tom20-mCherry even in the Mfn1/2-null MEFs (85.8 ± 1.4%) (Fig. 4a, Fig. S1). This shows that vMIA can traffic normally to mitochondria even in the absence of Mfn1 and Mfn2, and show that mitofusins are not required for vMIA trafficking to the OMM. FLIM analysis of vMIA-EGFP in Mfn1/2-null MEFs showed that vMIA-EGFP lifetime in Mfn1/2-null MEFs (τ = 3.10 ± 0.01 ns, n = 13) was significantly lower than the lifetime of cytosolic EGFP (τ = 3.46 ± 0.02 ns, n = 15, p < 0.0001) (Fig. 4b), and was similar to that of the WT-MEFs (Fig. 2). Thus, lack of mitofusins does not affect vMIA homodimerization.

Similar to our previous observation in primary HFFs and human cell lines^13,36^ and herein in MEFs (Fig. 1b,c), superresolution imaging by MSIM showed that vMIA is organized in nanoclusters at the OMM even in the Mfn1/2-null MEFs (Fig. 4c). Compared to the density of vMIA clusters at the OMM in WT MEFs (0.8 ± 0.05 clusters/µm, n = 50 mitochondria), Mfn1/2-null MEFs showed a 38% increase in the number of clusters (1.1 ± 0.05 clusters/µm/mitochondria, n = 40 mitochondria; p = 0.0005) (Fig. 4d, Fig. S2). Using MSIM imaging, we measured if this increase in the number of vMIA clusters affected the vMIA cluster size. This showed that despite the increased number of clusters/mitochondria, the vMIA cluster size was unaltered between the WT MEFs (179.9 ± 3.7 nm, n = 75 clusters) and Mfn1/2-null MEFs (177.1 ± 3.7 nm, n = 60) (Fig. 4e,f, Fig. S2). We found no difference in the percentages of mitochondria with clustered vMIA between the WT (91 ± 5.6%, n = 5) and Mfn1/2-null MEFs (87.5 ± 3.2%, n = 4) (Fig. S2). Thus, mitofusins are dispensable for the ability of vMIA to traffic to the OMM, and to homodimerize and form clusters at the OMM. However, the presence of mitofusins prevents the formation of excessive vMIA clusters at the OMM. An alternative possibility is that the increase in density of vMIA clusters at the OMM in Mfn1/2-null cells reflects the alteration of OMM protein distribution caused by the absence of mitofusins^26^.

### PACS-2 alters vMIA clustering without affecting vMIA trafficking to the OMM

**Figure 5:**
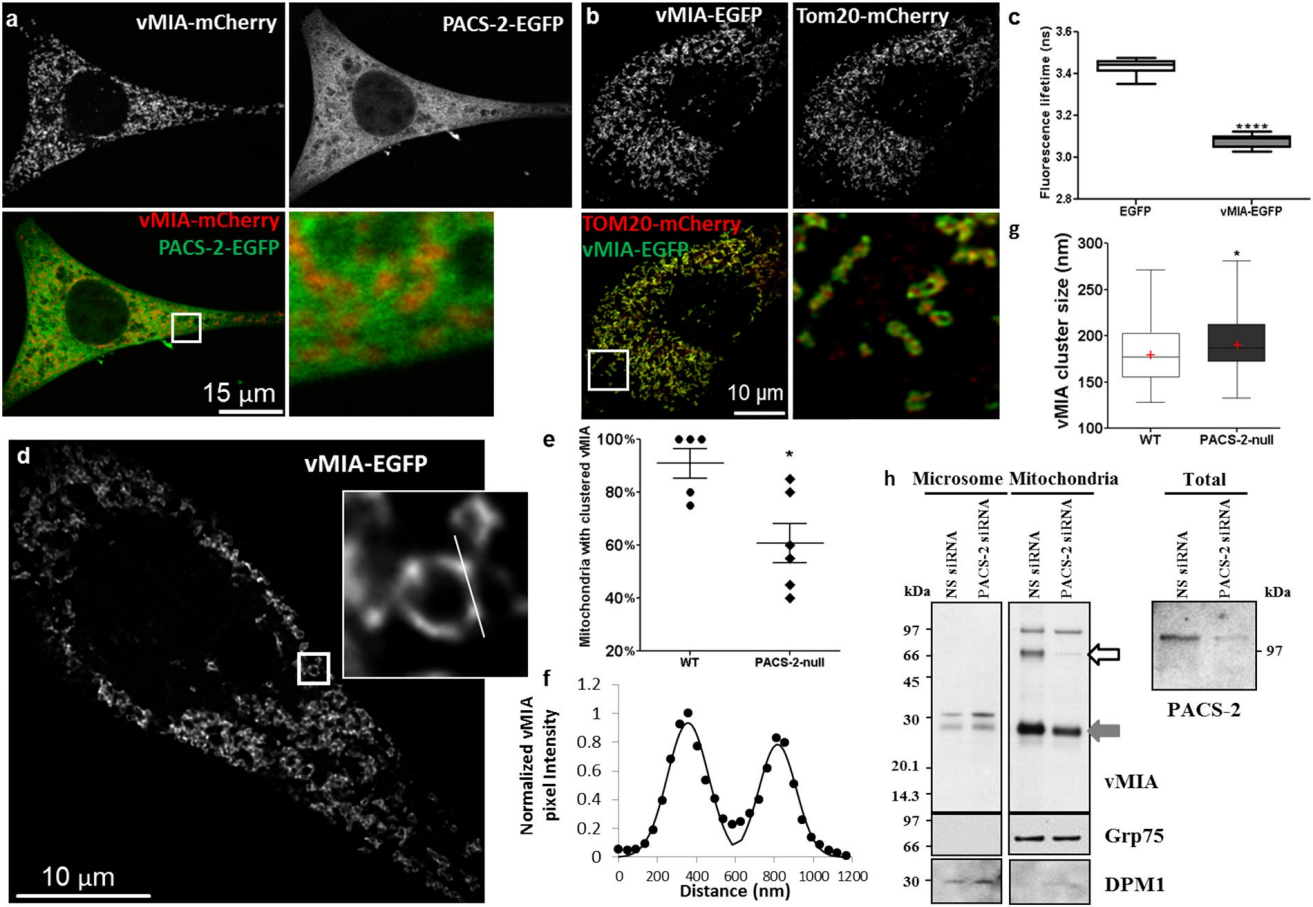
Role of PACS-2 in vMIA trafficking and clustering on the mitochondria. (**a**) WT MEFs transiently expressing vMIA-mCherry (red) and PACS-2-EGFP (green) were imaged by confocal microscopy. Shown are single confocal planes from a deconvolved z-stack as monochrome images of individual channels, which are pseudocolored for the merged image. The box marks the zoomed region. (**b**) A single confocal plane from a deconvolved z-stack of confocal images of PACS-2-null MEFs transiently expressing vMIA-EGFP (green) and Tom2-mCherry (red). The monochrome image of each fluorophore and the merged images are shown. The box marks the zoomed area. (**c**) The fluorescence lifetime comparison of cytosolic EGFP (τ = 3.44 ± 0.009 ns, n = 150 regions from 15 cells) and vMIA-EGFP (τ = 3.08 ± 0.009 ns, n = 120 regions from 12 cells) in PACS-2-null MEFs. (**d**) Zoomed single optical plane of a mitochondrion from a MSIM z-stack showing clustered distribution of vMIA-EGFP on the OMM of a transfected PACS-2-null MEF. (**e**) The percentage of mitochondria (20 mitochondria/cell) with clustered vMIA along the OMM in WT MEFs (n = 5 cells) and PACS-2-null MEFs (n = 6 cells). (**f**) Normalized pixel intensity of vMIA-EGFP along the line shown in the zoomed **d** inset. (**g**) Box plot showing the FWHM distribution of vMIA clusters (n = 60 clusters) in WT MEFs and PACS-2-null MEFs. The line and red, cross mark indicate the median and mean, respectively. (**h**) HeLa (PSS-1_20_) cells^8^ were lipofected with nonspecific (NS siRNA) or PACS-2 (PACS-2 siRNA) siRNAs and vector expressing WT vMIA and harvested 48 h later as described^18^. PACS-2 knockdown was assessed using rabbit anti-PACS-2 antiserum (1:500; gift from Dr. G. Thomas). Transfected cells were fractionated to obtain purified microsomes and mitochondria. 10 μg of fractionated proteins were separated by SDS-PAGE and analyzed by Western using anti-vMIA (DC35, 1:500)^8^ or mitochondrial (Grp75, 1:1000; Stressgen) marker. Monomeric and dimeric vMIA are indicated by the grey and open arrows, respectively. *Represents p < 0.05, ****represents p < 0.0001. The blots were cropped to enhance the conciseness of presentation. Full-length blots are presented in Supplementary Figure S5.

Another regulator of ER mitochondrial apposition is PACS-2^18^. Therefore, we examined if PACS-2-mediated ER-OMM apposition is required for vMIA trafficking from the ER to OMM. We tested the ability of vMIA-EGFP to traffic to the OMM in PACS-2-null MEFs. Transfection of control WT MEFs with vMIA-mCherry and PACS-2-EGFP showed that vMIA-labeled (red) mitochondria are interspersed between PACS-2-labeled (green) ER (Fig. 5a). vMIA-mCherry and PACS-EGFP distributed similarly in HFFs (Fig. S3). Similar to the WT MEF, vMIA-EGFP and Tom20-mCherry colocalized extensively in the PACS-2 null MEFs (88.4 ± 1.0%, n = 11 cells) (Figs 1a and 5b, Fig. S1). However, vMIA distributed more diffusely along the OMM in PACS-2-null MEFs, which increased colocalization of vMIA with Tom20 in PACS-2 null cells to 88.4 ± 0.8% (n = 11 cells) as compared to 83.9 ± 0.5% colocalization in the WT MEFs (p = 0.0006). This shows that vMIA traffics efficiently to mitochondria in PACS-2-null MEFs. FLIM analysis of vMIA-EGFP in PACS-2 null MEFs showed vMIA-EGFP lifetime of 3.08 ± 0.01 ns (n = 12 cells) in PACS-2-null MEFs, is similar to that in the WT MEF (τ = 3.10 ± 0.01) and is significantly lower than the corresponding PACS-2-null cells expressing cytosolic EGFP (τ = 3.44 ± 0.01 ns, n = 15 cells, p < 0.0001) (Fig. 5c).

By MSIM imaging of vMIA-EGFP in PACS-2-null MEFs, we found that while mitochondria with clustered vMIA can be detected (Fig. 5d), the percentage of such mitochondria with clustered vMIA (60.8 ± 7.5%, n = 6 cells) was lower than in WT MEFs (91.0 ± 5.7%, n = 5 cells, p = 0.04) (Fig. 5e, Fig. S2). These results show that vMIA’s ability to form clusters at the OMM is reduced by the reduced apposition between ER and OMM due to the lack of PACS-2. Concomitantly, the size (FWHM) of vMIA clusters was significantly larger in PACS-2-null MEFs (190.9 ± 3.7 nm, n = 60 mitochondria) as compared to the WT MEFs (179.9 ± 3.7 nm, n = 75 mitochondria, p = 0.02) (Fig. 5f,g, Fig. S2). However, the number of vMIA clusters/mitochondria was not different between the PACS-2-null (0.8 ± 0.05 clusters/µm) and WT MEFs (0.8 ± 0.06 clusters/µm) (Fig. S2). Together, above results with Mfn-null and PACS-2-null MEFs validate the importance of ER-OMM tethering on proper distribution and clustering of vMIA at the OMM.

To further verify the effects of PACS-2 on organization of vMIA at mitochondria, we used siRNA to knockdown PACS-2 in human (HeLa) cells (Fig. 5h). Using these cells transfected with vMIA and the biochemical fractionation approach we have developed to monitor ER to mitochondrial trafficking, we examined the role of PACS-2 in vMIA trafficking in human cells (Fig. 5h). Use of ER resident enzyme DPM1 and mitochondrial protein Grp75 as markers for ER and mitochondria, respectively, we verified the identity of these two fractions. As expected we observed vMIA in ER and mitochondrial fractions (Fig. 5h, filled arrow) and also observed vMIA dimers (Fig. 5h, open arrow). However, while vMIA monomers were also present in the ER and mitochondria fractions of PACS-2 depleted cells, the dimeric form was only detected in the mitochondrial fraction of cells treated with nonspecific siRNA but not with PACS-2 siRNA. These results independently validate our findings by superresolution imaging of the PACS-2 null MEFs that PACS-2 is not required for vMIA trafficking to the mitochondria, but is required for dimerization and proper organization of vMIA at the mitochondria. Use of both Mfn1/2-null and of PACS-2 null cells offers no evidence to support the bridge model for vMIA protein trafficking from ER to the mitochondria. Thus, we next tested the Drp1-mediated vesicular trafficking model for the ER-mitochondrial protein transport.

### Drp1 does not regulate vMIA trafficking and its clustering at the OMM

**Figure 6:**
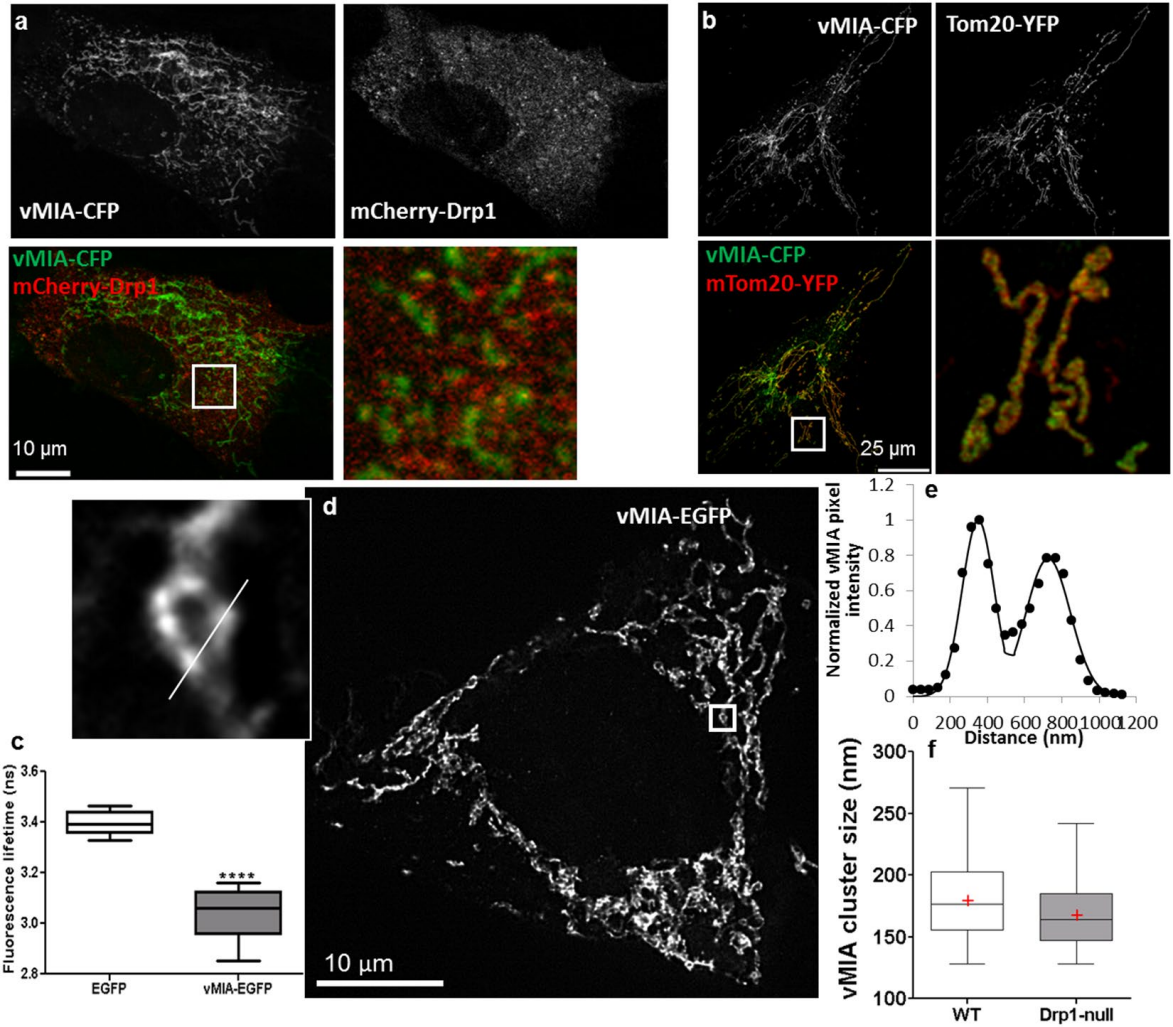
Role of Drp1 on vMIA trafficking and clustering on the mitochondria. (**a**) HFFs were lipofected with vectors expressing vMIA-CFP (pseudocolored green) and mCherry-Drp1 (red)^57^. Cells were imaged by confocal microscopy and single slice of the deconvolved images is shown as monochrome and pseudocolored images as above. (**b**) Confocal image of a Drp1-null MEF transiently expressing vMIA-CFP (pseudocolored green) and Tom20-YFP (pseudocolored red). Monochrome images show the individual channels and the merged image demonstrates vMIA colocalization with Tom20-YFP. A zoom of the boxed region of interest is shown. (**c**) The fluorescence lifetime comparison of cytosolic EGFP (τ = 3.40 ± 0.013 ns, n = 120 regions from 12 cells) and vMIA-EGFP (τ = 3.04 ± 0.026 ns, n = 130 regions from 13 cells) in Drp1-null MEFs. (**d**) A single plane from an MSIM z-stack images showing clustered distribution of vMIA-EGFP on the OMM of a transfected Drp1-null MEF. A zoom of a single mitochondrion shows vMIA clustering. (**e**) Normalized pixel intensities of a line shown along the mitochondrion in inset of panel d. (**f**) Box plot showing the distribution of vMIA clusters (n = 40 cells) in WT MEFs and Drp1-null MEFs. ****Indicates p < 0.0001.

Drp1 is known to associate with the ER and facilitate its normal morphology^20,47^, but its role in regulating vMIA-induced change in mitochondrial morphology has been debated^48,49^. However, Drp1 has been implicated in vesicular trafficking of AIF and Vpr from the MAM to mitochondria^4,10^. To test if Drp1 may facilitate vesicle-mediated trafficking of vMIA between ER and mitochondria, we used Drp1-null MEFs^41^. Using cells transfected with vMIA-CFP and mCherry-Drp1, we observed these two markers to colocalize in HFFs (mean, 65.9 ± 4.2%, n = 13 cells) and in WT MEFs (Fig. 6a, Figs S1 and S3). In Drp1-null MEFs, we observed efficient trafficking of vMIA to the mitochondria as shown by extensive colocalization of vMIA-CFP with Tom20-YFP (82.5 ± 2.8%, n = 11 cells) (Fig. 6b), which is similar to the WT MEFs (83.9 ± 0.5%) (Fig. S1). Thus, vMIA can traffic efficiently to mitochondria even in the absence of Drp1-mediated vesicular trafficking between ER and mitochondria. FLIM analysis of vMIA-EGFP showed that the lack of Drp1 did not affect the ability of vMIA to homodimerize (Fig. 6c) - vMIA-EGFP lifetime (τ = 3.04 ± 0.03 ns, n = 13 cells) was significantly lower than the cytosolic EGFP (τ = 3.40 ± 0.01 ns, n = 12 cells, p < 0.0001), and no different from that of vMIA-EGFP lifetime in WT MEFs (Fig. 2e).

MSIM imaging of vMIA-EGFP demonstrated the ability of vMIA to form clusters at the OMM in Drp1 null cells (Fig. 6d–f). These vMIA-EGFP clusters in Drp1-null cells were similar in size (172.1 ± 2.8 nm; n = 100 mitochondria) to those observed in WT MEFs (179.9 ± 3.7 nm; n = 75 mitochondria) (Fig. 6e,f, Fig. S2). Compared to the WT MEFs, lack of Drp1 affected neither the number of vMIA clusters/µm (0.8 clusters/µm/mitochondria) nor the percentage of mitochondria with clustered vMIA (100 ± 0%) (Fig. S2). Thus, by all the measures used, we found that lack of Drp1-mediated vesicle trafficking did not affect the ability of vMIA to traffic from ER to mitochondria, dimerize, or properly cluster at the OMM.

### Lipid and hydrophobic interactions reduce vMIA dimerization and clustering at the OMM

Above results identify that protein-mediated ER-OMM tethering regulates the ability of vMIA to form clusters at the OMM, but does not affect ER to OMM protein trafficking. Through the use of two vMIA mutants that have leader sequence with higher hydrophobicity - high hydrophobicity A (HHA) and B (HHB) mutants we have previously identified that hydrophobic interactions of vMIA affect its trafficking to the mitochondria^11^. Between the vMIA hydrophobicity mutants, HHA and HHB, it is the HHB mutant that has larger hydropathy score of its N-terminal leader (residues 1–22)^11^. In contrast, the vMIA cholesterol binding domain II (CBDII) mutant has a mutant cholesterol binding domain (residues 14–23), which reduces its association with the MAM lipid rafts, without affecting trafficking to the OMM^32^. Using these two vMIA mutants with defects in lipid binding properties and divergent trafficking fates, we assessed if altered lipid interactions affect vMIA’s ability to homodimerize and form clusters.

**Figure 7:**
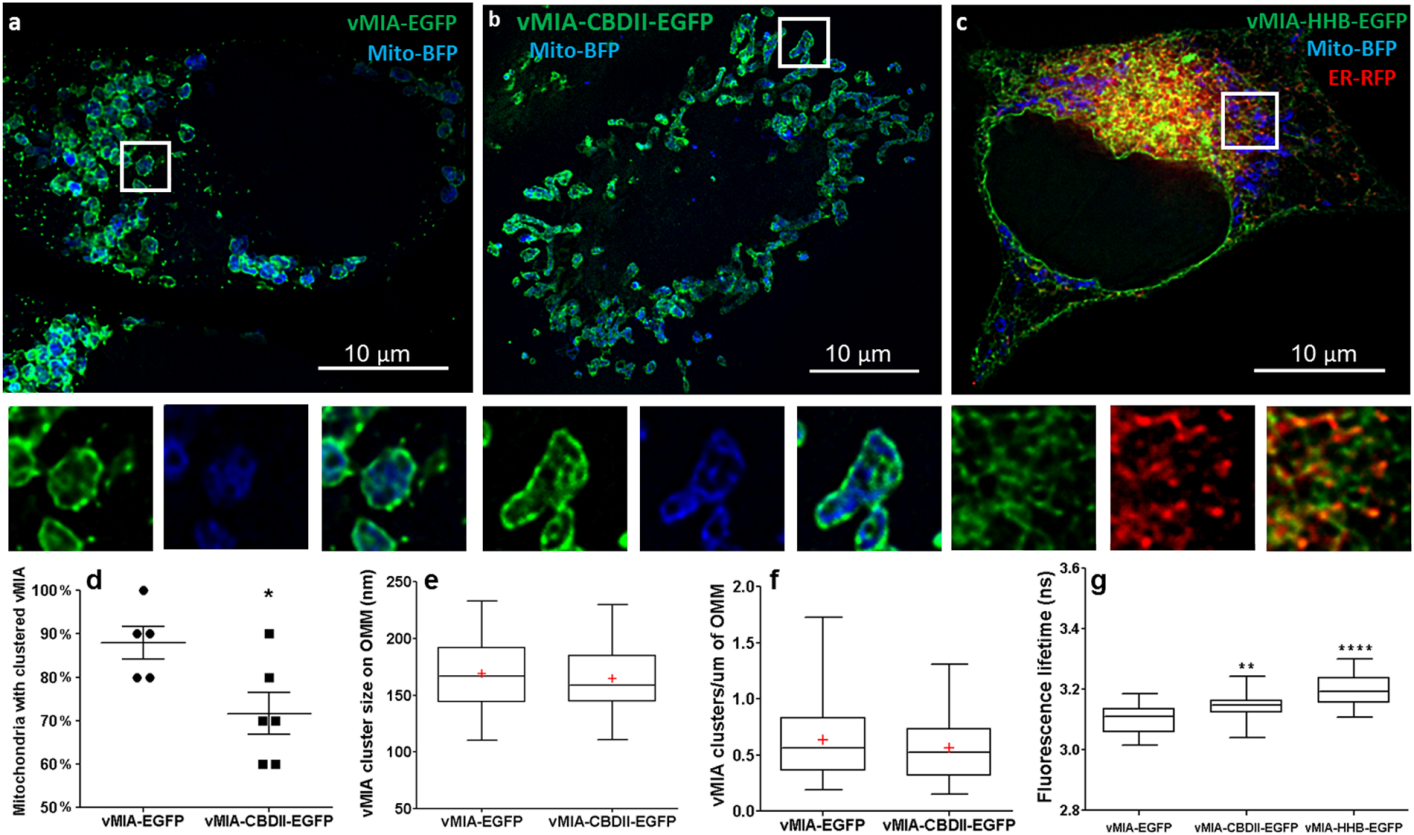
Analyses of vMIA HHB and CBDII mutant clustering by MSIM and FLIM. HeLa cells were transiently transfected with vectors expressing (**a**) vMIA-EGFP, (**b**) vMIA-CBDII-EGFP or (**c**) vMIA-HHB-EGFP (green) as previously described^36^. The cells were co-transfected with only a mitochondrial marker (Mito-BFP, blue) (**a**,**b**), or with Mito-BFP and an ER marker (ER-RFP, red) (**c**). Cells were fixed and a single plane from an MSIM z-stack image showing distribution of vMIA-EGFP and mitochondrial and ER marker is shown. A zoom of regions marked by the white boxes in each image is shown below. (**d**) The percentages of mitochondria with clustered vMIA in HeLa cells expressing vMIA-EGFP (88.0 ± 3.7%, n = 5 cells) or vMIA-CBDII-EGFP (71.7 ± 4.8%, n = 6 cells). (**d**) The size of vMIA (mean 169.18, min: 110, median 167, max: 233, n = 50) and vMIA-CBDII (mean 164.55, min: 111, median 159, max: 230, n = 60) clusters were determined. p = 0.4024. The lines and red crosses indicate the medians and means, respectively. (**f**) The number of vMIA clusters in HeLa cells expressing vMIA-EGFP (mean 0.638, min: 0.194, median 0.562, max: 1.73, n = 50) or vMIA-CBDII-EGFP (mean = 0.563, min: 0.153, median 0.526, max: 1.312, n = 60) were determined (p = 0.3118). The line and red, cross mark indicate the median and mean, respectively. (**g**) vMIA-HHB and vMIA-CBDII are defective in dimerization as measured by FLIM. Shown are the fluorescence lifetime comparisons of vMIA-EGFP (τ = 3.10 ± 0.009 ns), vMIA-CBDII-EGFP (τ = 3.14 ± 0.007 ns) and vMIA-HHB-EGFP (τ = 3.2 ± 0.008 ns). *Represents p < 0.03, **represents p < 0.003, ****represents p = 0.0001.

Using FLIM, we assessed the ability of vMIA and its mutants to homodimerize. Compared to the WT vMIA-EGFP (τ = 3.10 ± 0.01 ns), lifetimes of both the mutant vMIA-EGFP were increased - vMIA-CBDII-EGFP (τ = 3.14 ± 0.01 ns; p = 0.003) and vMIA-HHB-EGFP (τ = 3.2 ± 0.01 ns; p < 0.0001) (Fig. 7g). Using MSIM imaging, we readily detected clustering of WT vMIA and of vMIA CBDII at the OMM (Fig. 7a and b, Fig. S4). In contrast, vMIA HHB mutant localized away from the mitochondria, on the ER and did not form clusters (Fig. 7c). Further, compared to WT vMIA, which formed clusters on 88.0 ± 3.7% (n = 5 cells) mitochondria, vMIA CBDII mutant formed clusters in fewer mitochondria (71.7 ± 4.8%, n = 6 cells; p = 0.03) (Fig. 7d). Despite the reduced homodimerization and reduction in the number of mitochondria with vMIA-CBDII clusters, neither the vMIA-CBDII cluster sizes (Fig. 7e) nor the cluster density per mitochondria (Fig. 7f) were different from WT vMIA. Together, these results show that lipid and other hydrophobic interactions facilitate ER-OMM trafficking and the nanoscale organization of vMIA at the OMM.

## Discussion

Through the use of multiple available MEF knockout cell lines, we have performed a comprehensive analysis of ER-mitochondrial trafficking and OMM organization of signal-anchored viral protein vMIA. Our approach of using a combination of mouse (MEFs) and human (HeLa cells and HFFs) cells to study vMIA trafficking is based upon the previous findings from multiple laboratories that vMIA traffics efficiently and similarly in these CMV permissive and non-permissive cells^7–9,11,13,27,32,36,43,50,51^. Furthermore, most of vMIA functions (including mitochondrial fragmentation, actin rearrangement, host cell protein retargeting to mitochondria, metabolic effects, inhibition of ATP synthesis, and inhibition of antiviral signaling) are unaffected by the cell being permissive or not to CMV growth^27,43,51^. Our results herein show that Mfn1, Mfn2, and PACS-2-mediated ER-mitochondria tethering is not required for ER-mitochondria trafficking. Instead, we show that these proteins facilitate clustering of proteins at the OMM. Further, we find that lack of Drp1 does not affect ER to mitochondrial trafficking or OMM clustering of vMIA. While Drp1 has been implicated in vesicle mediated trafficking between ER and mitochondria^4,10^, it is conceivable that lack of Drp1 resulting in elongated mitochondria may also impact on ER-mitochondria tethering. However, our observation that Drp1 deletion did not affect vMIA trafficking or clustering at the mitochondria suggests a potential role of Drp1 in mediating ER-mitochondria tethering is also not relevant for vMIA trafficking or clustering at the OMM. Mfn1/2 and PACS-2 have been shown to maintain ER-mitochondria apposition^18,21^.

vMIA interacts with Mfn2^45^, while PACS-2 is known to alter subcellular distribution of the cellular MAM protein calnexin^22^. Despite these results suggesting a potential involvement of Mfn1/2 and PACS-2 in vMIA trafficking from the ER to mitochondria, we find this is not the case. We thus envision that vMIA may traffic to mitochondria by multiple, parallel pathways for trafficking from the ER to mitochondria, such that knock out of any one pathway does not inhibit its trafficking but affects its optimal organization at the OMM. Alternatively, vMIA may traffic by a pathway that is independent of ER-mitochondrial tethering by PACS-2 and Mfn2 and of vesicle formation by Drp1. Such a pathway may use lipid affinity or a cytosolic lipid transport protein to translocate without vesicular or bridging requirements. It was recently found that phosphatidylserine (PS) transport from the ER to mitochondria does not require proteinaceous linkers between the organelles but a cytosolic protein, VAT-1, which has affinity for PS^52^. In favor of such a mode of trafficking, we found that vMIA mutant that has increased hydropathy fails to traffic to the OMM^11^.

vMIA’s ability to translocate to the lipid-synthetic rich MAM subdomains potentially enables it to usurp lipid trafficking pathways. Association with lipids is known to enable some proteins to target the MAM. For example, Sig-1R protein utilizes cholesterol affinity to associate with the MAM^31^. Palmitoylation enriches calnexin and TMX in the MAM^29^. Indeed, lipid association appears to enable vMIA trafficking from the MAM to mitochondria. We found that the vMIA HHA and HHB mutants, which have increased hydrophobicity of the N-terminal leader, are retargeted to the secretory apparatus and are less efficient in mitochondrial trafficking^11^. These results suggest that vMIA’s targeted association with lipids in the ER/MAM subdomain, in part, underlies its ability to translocate to the OMM. In addition, other vMIA MTS elements, including modification of its ^21^SY motif and downstream proline rich domain (^33^PLPP), regulate its trafficking to the OMM^11^. Nonetheless and in contrast to Sig-1R, vMIA targeting to the MAM and mitochondria is not mediated by cholesterol binding. vMIA CBD mutants, defective in cholesterol binding, traffic efficiently from the MAM (but not to the MAM lipid rafts) to mitochondria^32^. Once in the MAM lipid synthetic enriched sub-domain, we suggest that vMIA could use a cytosolic lipid carrier to translocate to the OMM as has been shown for PS trafficking from the outer to inner leaflet of the mitochondrial membrane, by the cytosolic protein VAT-1^52^.

Following trafficking to the mitochondria, vMIA forms nanometric clusters in the OMM^13,36^. This organization of vMIA clusters at the OMM is similar to that of multiple mitochondrial proteins^33,35,37^. However, the mechanism underlying clustering of these proteins has not been addressed. Our results show that Drp1 does not affect the ability of vMIA to homodimerize, but PACS-2 and mitofusins are needed for proper distribution and clustering of vMIA at the OMM. The density of vMIA clusters at the OMM appears to be negatively regulated by Mfn1/2 such that in their absence, more vMIA clusters are localized in the OMM. This may result from vMIA’s ability to interact with Mfn1/2^45^. Herein, we documented that vMIA redistributes Mfn2 at the OMM. This interaction with and redistribution of Mfn2 may exclude interactions with cellular proteins present in vMIA clusters and thereby reduce vMIA clustering in presence of Mfn1/2. In contrast, the absence of PACS-2 reduces the percentage of mitochondria with clustered vMIA. As PACS-2 regulates apposition of the ER and OMM, its presence may enhance the number of MAM contact sites which may, in turn, affect vMIA distribution and clustering in OMM subdomains close to the MAM tethering sites.

Emerging evidence suggests that mitochondrial lipids can modulate the organization and distribution of some mitochondrial nanoscale complexes - low ergosterol content of yeast mitochondrial membranes for targeting of some OMM proteins with C-terminal anchors^53^. Consistent with this, we find that the cholesterol binding mutant of vMIA (CBD II) reduced the number of mitochondria with clustered vMIA. Moreover, in addition to sterol, the mitochondria specific lipid cardiolipin has been shown to be required for the localization of yeast Mic27/Mic10/Mic12 sub-complex at cristae junctions in the inner mitochondrial membrane^54^. These roles of lipids suggest the possibility that the lipid composition of the MAM and of the OMM facilitate ER to mitochondrial trafficking of vMIA through association with its leader sequence. Our work highlights the need to study the role of lipids in ER-mitochondria targeting and OMM organization of proteins.

## Methods

### Cell culture and transfection

HFFs (Viromed Laboratories) were grown in Dulbecco’s Modified Eagle’s medium (DMEM) supplemented with 10% fetal calf serum (FCS), 100 U/ml of penicillin, 100 µg/ml of streptomycin and 2 mM of L-glutamine at 37 °C and 5% CO_2_. WT MEFs, Mfn-null MEFs (Mfn1 −/−, Mfn2 −/−)^40^, and PACS-2-null (PACS-2 −/−) MEFs^39^ were cultured in DMEM supplemented with 10% FCS, 100 U/ml of penicillin and 100 µg/ml of streptomycin; whereas, Drp1-null MEFs (Drp1 −/−)^41^ were cultured in Iscove’s Modified Dulbecco’s Medium (IMDM) supplemented with 10% FCS, 100 U/ml of penicillin and 100 µg/ml streptomycin.

HFFs and MEFs were plated on 18 mm (for confocal microscopy) or 25 mm (for MSIM) cover slips and transfected one day later using Lipofectamine 2000 (Life Technologies) as previously described^36^. Plasmids encoding vMIA-EGFP^55^, vMIA-CFP^11^, mouse Tom20 (Tom20)-YFP^56^, PACS-2-EGFP^18^, mCherry-Drp1^57^, Mfn2-YFP^48^, Tom20-mCherry, or vMIA-mCherry were used for transfection. Monomeric EGFP expression vector (Clontech Laboratories, Madison WI) was used as a control.

Tom20-mCherry expression plasmid was generated by sub-cloning of the mouse Tom20 cDNA upstream of the mCherry open reading frame in mCherry N-1 vector (Clontech Laboratories). The vMIA open reading frame was analogously sub-cloned into pmCherry N-1. The plasmid sequence was confirmed using a commercial sequencing company (Macrogen).

### PACS-2 siRNAs

PACS-2 siRNAs^18^ were used as described to reduce PACS-2 expression and destabilize ER-mitochondria contacts. A nonspecific random siRNA (5′-CGUUUGCGGUGUUUAUGGCtt-3′; 5′-GCCAUAAACACCGCAAACGtt-3′) was used as a control. BLASTN searches with the nonspecific siRNA detected no significant similarity in the human genome database (A. Colberg-Poley, unpublished results). Briefly, HeLa cells expressing EGFP tagged human phosphatidylserine synthase 1, named PSS-1_20_ cells^8^, were transfected with 7 μg of vMIA expression vector and 100 nM of PACS-2 siRNAs (Dharmacon) or 100 nM of nonspecific siRNAs (Ambion).

### Fractionation of mitochondria and microsomes

Mitochondria and microsomes were isolated from transfected PSS-1_20_ cells as described^8,58^. Briefly, cells were washed twice in PBS and pelleted by centrifugation, resuspended and lysed using a motor-driven Potter-Elvehjem homogenizer. The supernatant from pelleted homogenates was then centrifuged at 10,300 × *g* to separate microsomal (microsomes and cytosol) from crude mitochondrial (MAM and mitochondria) fraction. Microsomes were pelleted by ultracentrifugation at 100,000 × *g* from the crude microsomal fraction (supernatant). Mitochondria fractions were separated in self-generating Percoll gradients. Isolated fractions were diluted fivefold in sucrose homogenization medium and subjected separately to a centrifugation at 6,300 × *g* for 10 min at 4 °C. The pellet of the mitochondria centrifugation was used as the purified mitochondria in our analysis. All of the fractions were resuspended in sucrose homogenization medium and analyzed by Western blot analysis.

### Confocal microscopy and analysis

Confocal images were acquired on Olympus FV 1000 and Leica TCS SP8 microscopes. On the Leica TCS SP8, a HC PL APO CS2 100x/1.40 Oil objective was used to acquire images. 514 nm and 633 nm laser lines from a White Light Laser was used to excite YFP and Alexa 647 fluorophores. The emission was collected on hybrid detectors with the AOBS set to 520–610 nm and 640–750 nm respectively. These images were deconvolved using the Huygens software (Scientific Volume Imaging). On the Olympus FV 1000, UPlanSApo 100 X/1.40 NA oil objective was used to acquire 1024 × 1024 pixels image, where each pixel was 62 nm in size. A step size of 410 nm was used for the z-stacks. For images that were to be deconvolved, step size of 110 nm was used for acquiring the z-stacks. 440 nm, 488 nm, 515 nm and 559 nm lasers were used to excite CFP, GFP, YFP and mCherry fluorophores, respectively. For triple color (CFP-YFP-mCherry) imaging 405 nm laser was used to excite CFP and emission collected with (425–475 nm filter), which prevented any excitation or emission bleedthrough and optimal excitation of YFP and mCherry. The image analysis was carried out using Olympus Fluoview version 4.0, MetaMorph Premier and Fiji ImageJ software. For image deconvolution CellSens Dimension software version 1.12 (Olympus Life Science) was used. Constrained iterative 3-D deconvolution module with the advanced maximum likelihood estimation algorithm (ADVMLE) was employed. The plots used to identify clustering on the deconvolved images were made using MetaMorph Premier (7.7.0) software, which was provided by Molecular Devices, LLC. For pixel colocalization analysis the individual channels of the confocal images were separated and the average intensity of pixels within the selection was calculated. The resulting value of 0.9 X average intensity was used to threshold the channel. Integrated morphometry analysis (IMA) module of the MetaMorph Premier 7.0 software was used to identify objects in the image larger than 0.14 µm^2^ to generate the mask for use in colocalization analysis.

### Gated Stimulated Emission Depletion Microscopy (GSTED)

STED imaging was done using the Leica TCS SP8 equipped with a White Light Laser, two depletion lasers and hybrid detectors. A HC PL APO CS2 100x/1.40 Oil objective was used to acquire 12-bit images with pixel size less than 25 nm. The Tom20-Alexa 647 was excited at 633 nm and depleted with 775 nm laser. The emission was collected between 640 nm to 750 nm with a time gating of 0.3 ns to 6.0 ns. Sequential stack for vMIA-YFP, using a 514 nm excitation and 660 nm depletion, was acquired from 520 nm to 610 nm and time-gated between 1.5 ns and 6.0 ns. Z-stacks with a step size of 170 nm or less was acquired and deconvolved using the Huygens Professional software (Scientific Volume Imaging).

### MSIM Sample Preparation and Imaging

At 16 to 24 hours post-transfection, cells were fixed using 4% paraformaldehyde (PFA) (Electron Microscopy Sciences) in PBS, pre-warmed to 37 °C, for 15 minutes at room temperature. After fixation cells were washed with PBS and coverslips were mounted with ProLong Diamond Antifade Reagent (Life Technologies) onto slides and left overnight to dry at room temperature. For MSIM imaging, tetraspecs (Thermo Fisher) were added onto 25 mm coverslips that contained cells with multiple fluorophores prior to mounting onto another 25 mm coverslip with ProLong Diamond Antifade Reagent and left to dry overnight at room temperature.

The MSIM microscope used for these experiments is made from an Olympus IX-71 widefield microscope as previously described^36^. Briefly, critical modifications made to provide an increase its resolution capabilities include placing a microlens array in the illumination pathway to provide a two-dimensional pattern of excitation spots within the focus of the objective lens. This pattern was translated in approximately 1 pixel size steps over the field of view while collecting an image at each position. Images were taken in z-stacks with 200 images taken per plane for each fluorophore used. MSIM processing was performed using code written in Python and provided by the Hari Shroff lab at the NIH^59^. After processing, the resulting images were a widefield image, and deconvolution of the widefield, and the MSIM image. MSIM images taken were 512 × 512 pixels; however, during processing, images were scaled up to 1024 × 1024 pixels. Any further processing of the MSIM images was done in ImageJ (NIH). Light sources used to excite fluorophores within cells were a 50 mW 488 laser to illuminate green fluorophores and a 50 mW 561 Sapphire laser to illuminate red fluorophores. All settings for the microscope were controlled using Micro-Manager. A 100 × 1.4 NA Nikon oil immersion objective was used for imaging in these experiments.

### FLIM

The frequency domain FLIM analysis was performed with live cells roughly 20 hours after transfection. An Olympus BX81 inverted microscope was used to acquire widefield FLIM images. All the images were acquired using a UApoN 100 X/1.49 oil objective. A 488 nm laser (Intelligent Imaging Innovations) modulated at 50 MHz was used to excite the fluorophores in the samples. The fluorescence lifetime signal was measured using the II18MD modulated image intensifier (Lambert Instruments). The modulation was set to 0.23 and the intensification was set to 2,500. A CoolSNAP EZ camera (Photometrics) was used to acquire phase-shifted images. Rhodamine 101 (Sigma-Aldrich) dissolved in water, with a lifetime of 4.32 ns, was used to calibrate the system. It was also used to check the stability of the system over the course of the experiment. Images from 10–15 fields of view were collected for each sample type. At least 10 regions of interest (ROI) were marked randomly on each cell. The fluorescence lifetime from these ROIs were analyzed. The image acquisition and analysis was done using Slidebook version 6 software from Intelligent Imaging Innovations. The lifetime of individual pixels was calculated using the polar plot representation for the frequency-domain fluorescence lifetime data^60^.

### Statistical analysis

The methods for data acquisition and the sample sizes used are described for each individual experiment. Quantitative data are expressed as the mean value (mean ± standard error of the mean (SEM)). Statistical analysis of all the experimental data was performed using GraphPad Prism 5 software. Each set of values were tested for normality to choose appropriate statistical analysis. The values for vMIA mutant cluster sizes (WT and CBDII) and for vMIA colocalization with mitochondria were normally distributed and thus two-tailed unpaired t test was used for p-value measurements. For all the other analyses the data were tested using two-tailed Mann-Whitney U t-test.

## Electronic supplementary material

Supplementary Figures and Figure Legends
