# Supplementary material for: Superresolution Imaging Identifies That Conventional Trafficking Pathways Are Not Essential for Endoplasmic Reticulum to Outer Mitochondrial Membrane Protein Transport
Source: Sci Rep. 2017 Feb 2;7:16. doi: 10.1038/s41598-017-00039-5 (PMC5428351; doi:10.1038/s41598-017-00039-5)
Supplement: Supplementary file 1 — Supplementary Figures and Figure Legends [file 41598_2017_39_MOESM1_ESM.pdf]

# Viral Mitochondria-localized Inhibitor of Apoptosis Encoded by Cytomegalovirus Bypasses Conventional Pathways to Traffic from Endoplasmic Reticulum to Outer Mitochondrial Membrane

Kyle Salka[1], Shivaprasad Bhuvanendran[1], Kassandra Wilson[1], Petros Bozidis[1,2], Mansi Mehta[1], Kristin Rainey[3], Hiromi Sesaki[4], George H. Patterson[3], Jyoti K. Jaiswal[1, 5, *], and Anamaris M. Colberg-Poley[1, 5, 6, *]

## Supplementary Information

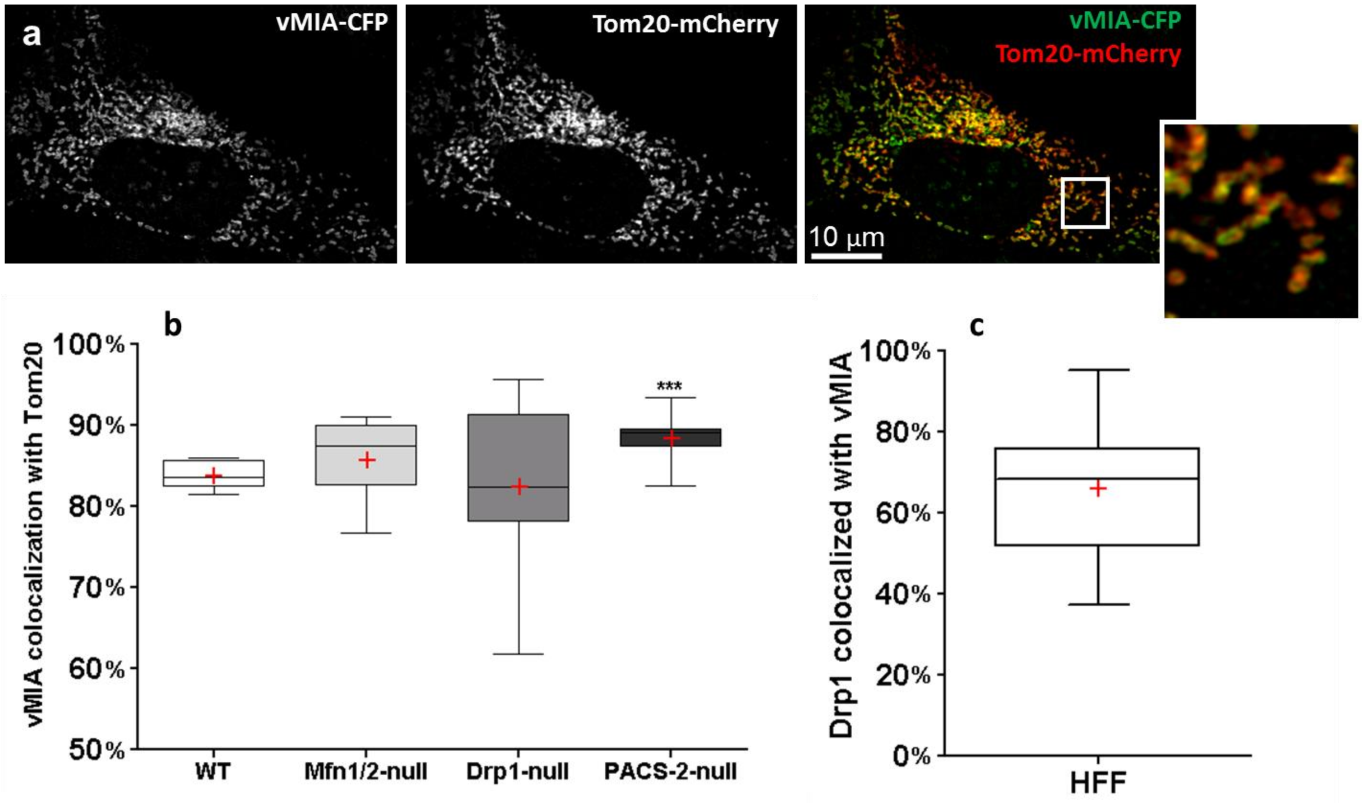


**Figure S1. Analysis of vMIA Colocalization with Cellular Proteins.** (**a**) WT MEFs were transfected to express vMIA-CFP (pseudocolored green) and the OMM marker, Tom20-mCherry (red) transiently (19 hours). The cells were then imaged by confocal microscopy and shown here are individual channels (gray scale) and the merged image of a single confocal plane from a deconvolved z-stack. The inset shows the zoom of a boxed area. (**b**) WT MEFs, Mfn1/2-null, Drp1-null, and PACS-2-null were lipofected with vectors expressing vMIA-CFP and mTom20-mCherry (WT and Mfn1/2-null), vMIA-CFP and mTom20-YFP (Drp1-null), or vMIA-EGFP and mTom20-mCherry (PACS-2-null). Cells were fixed with 4% PFA at 19 hours after transfection and imaged with an Olympus FV1000 confocal microscope using a 100 X objective. Colocalization of the masked images (mitochondrial channel and vMIA channel) was measured using MetaMorph and presented as percent area overlap between the two channels. (**c**) HFFs were transiently transfected with plasmids expressing vMIA-CFP and mCherry-Drp1 and in Fig. **S3**. Colocalization analyses of mCherry-Drp1 with vMIA-CFP were performed using MetaMorph. The mean and median colocalization of mCherry-Drp1 with vMIA-CFP is 65.9 and 68.2%, respectively, and ranges from 37.4%-95.0%, n = 13. The lines and red crosses indicate the medians and means, respectively.

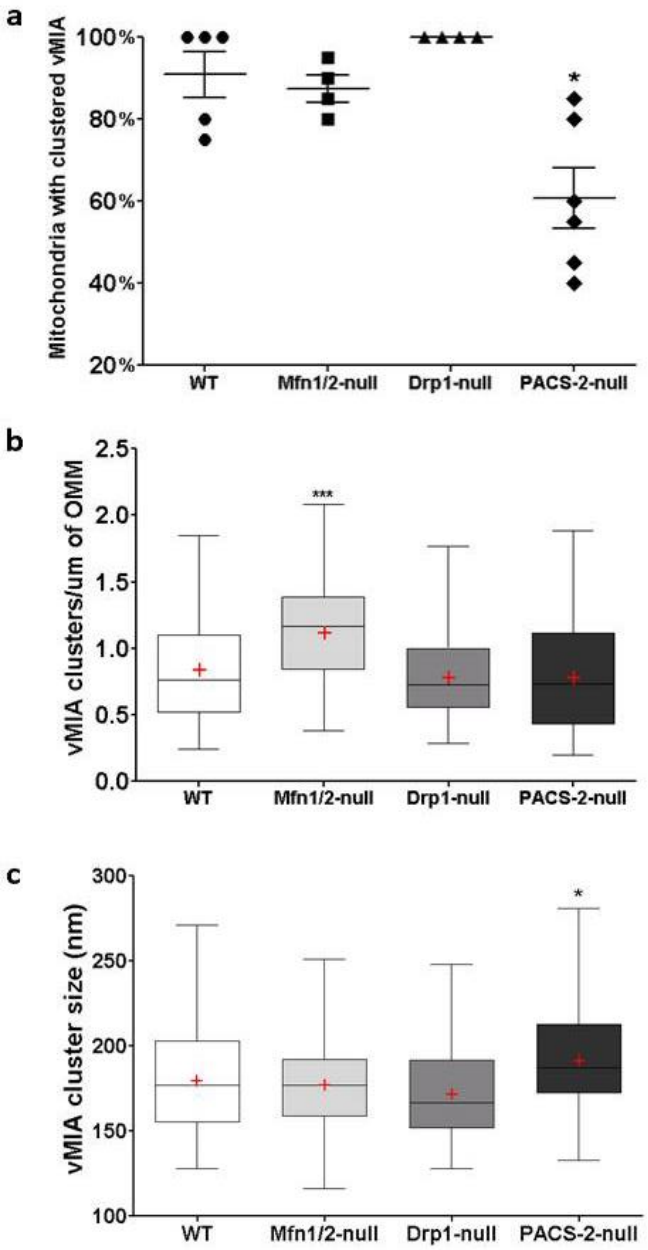


**Figure S2. Analysis of vMIA Clustering at the OMM.** (**a**) The numbers of mitochondria in WT, Mfn1/2-null, Drp1-null and PACS-2-null MEFS with clustered vMIA were determined. Indicated are the means (center lines) and S.E.M. (whiskers). The percentage of mitochondria with clustered vMIA for WT MEFs is 91 $\pm$ 5.6%, (n = 5 cells), for Mfn1/2-null cells is 87.5 $\pm$ 3.2%, (n = 4 cells) and for Drp1-null cells is 100 $\pm$ 0%, (n = 4). Compared to the WT MEFs, the percentage of mitochondria with clustered vMIA in PACS-2-null MEFs was reduced to 60.8 $\pm$ 7.5%, (n = 6; p = 0.04). (**b**) For mitochondria that show clustered vMIA distribution, the number of vMIA clusters per unit length of mitochondrial perimeter was determined in WT, Mfn1/2-null, Drp1-null, and PACS-2-null MEFs. Shown are the medians (center lines, 0.76), range

(0.24-1.85, whiskers) and average (0.84; red mark) of vMIA cluster density in WT (n = 50), Drp1-null (median = 0.73, mean = 0.78, range 0.28 - 1.77, n = 40), and PACS-2-null (mean 0.79, median 0.73, range 0.20 - 1.89, n = 60) MEFs. The density of vMIA clusters in Mfn1/2-null MEFs was larger (mean = 1.123, median = 1.161, range = 0.38-2.1, n = 40, ***p-value = 0.0005) than in WT MEFs. The lines and red crosses indicate the medians and means, respectively. (**c**) The sizes of the vMIA clusters were measured using a single MSIM z-stack plane and FWHM values of vMIA clusters were measured over indicated number of mitochondria. The plots show the means (cross mark), medians (center line), and the whiskers represent the size ranges. vMIA cluster sizes were determined in WT (mean = 179.9 nm, median = 177.0 nm, range = 128.0 - 271.0 nm, n = 75), Mfn1/2-null (mean = 177.1 nm, median = 177.0, range =116.0 - 251.0 nm, n = 60), Drp1-null (mean = 172.1, median = 166.5, range = 128.0 - 248.0 nm, n = 100). vMIA cluster sizes for PACS-2-null MEFs (mean = 190.9 nm, median = 187.0, range = 133.0 - 281.0 nm, n = 60) were significantly greater than the WT MEFs (p = 0.02).

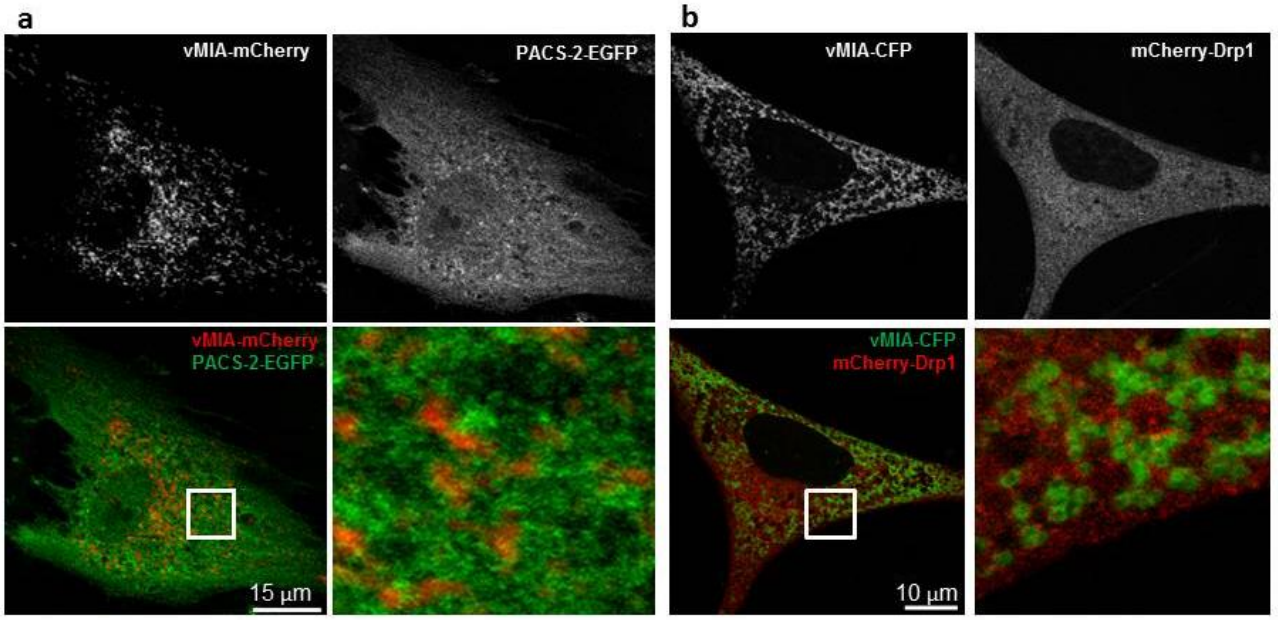


**Figure S3. Colocalization of vMIA with PACS-2 and Drp1 in HFFs.** HFFs transfected with plasmids expressing (**a**) vMIA-mCherry (red) and PACS-2-EGFP (green) and (**b**) vMIA-CFP and Drp1-mCherry were imaged by Olympus FV1000 confocal microscope with a 100 X objective. The monochrome image of each fluorophore is shown together with the pseudocolored merged image. The white box on the merged image shows the region of interest that is zoomed in the adjacent image.

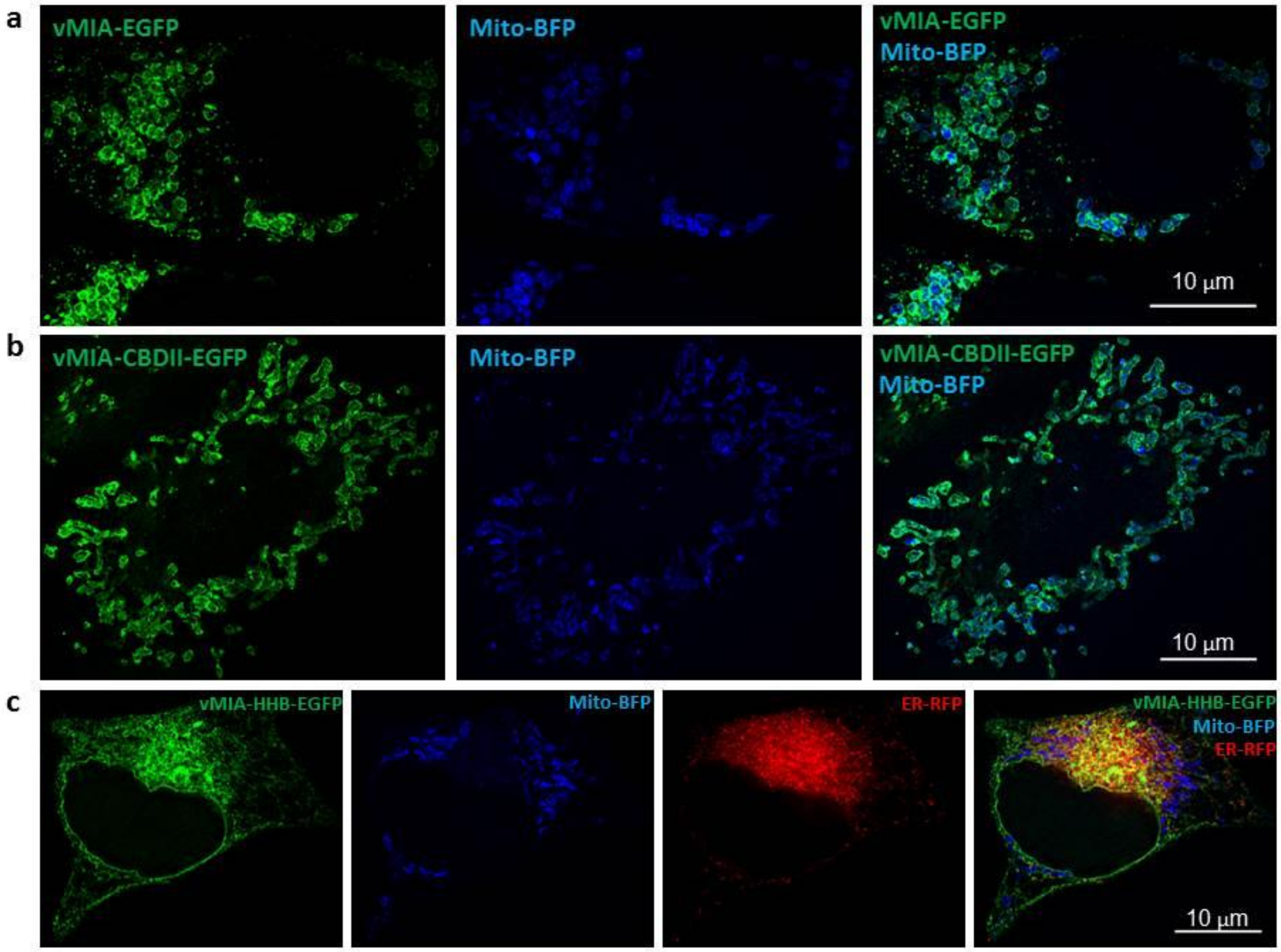


**Figure S4. Individual channels for the MSIM images of WT vMIA, vMIA-CBDII, and vMIA-HHB Shown in Fig. 7.** HeLa cells were transiently transfected with vectors expressing (**a**) vMIA-EGFP, (**b**) vMIA-CBDII-EGFP, or (**c**) vMIA-HHB-EGFP and imaged as described in Fig. 7. Shown are the individual channels for vMIA-EGFP (**a**), vMIA-CBDII-EGFP (**b**), and vMIA-HHB-EGFP (**c**) (green), Mito-Blue (**a, b, c**) (blue), and ER- RFP (**c**, red).

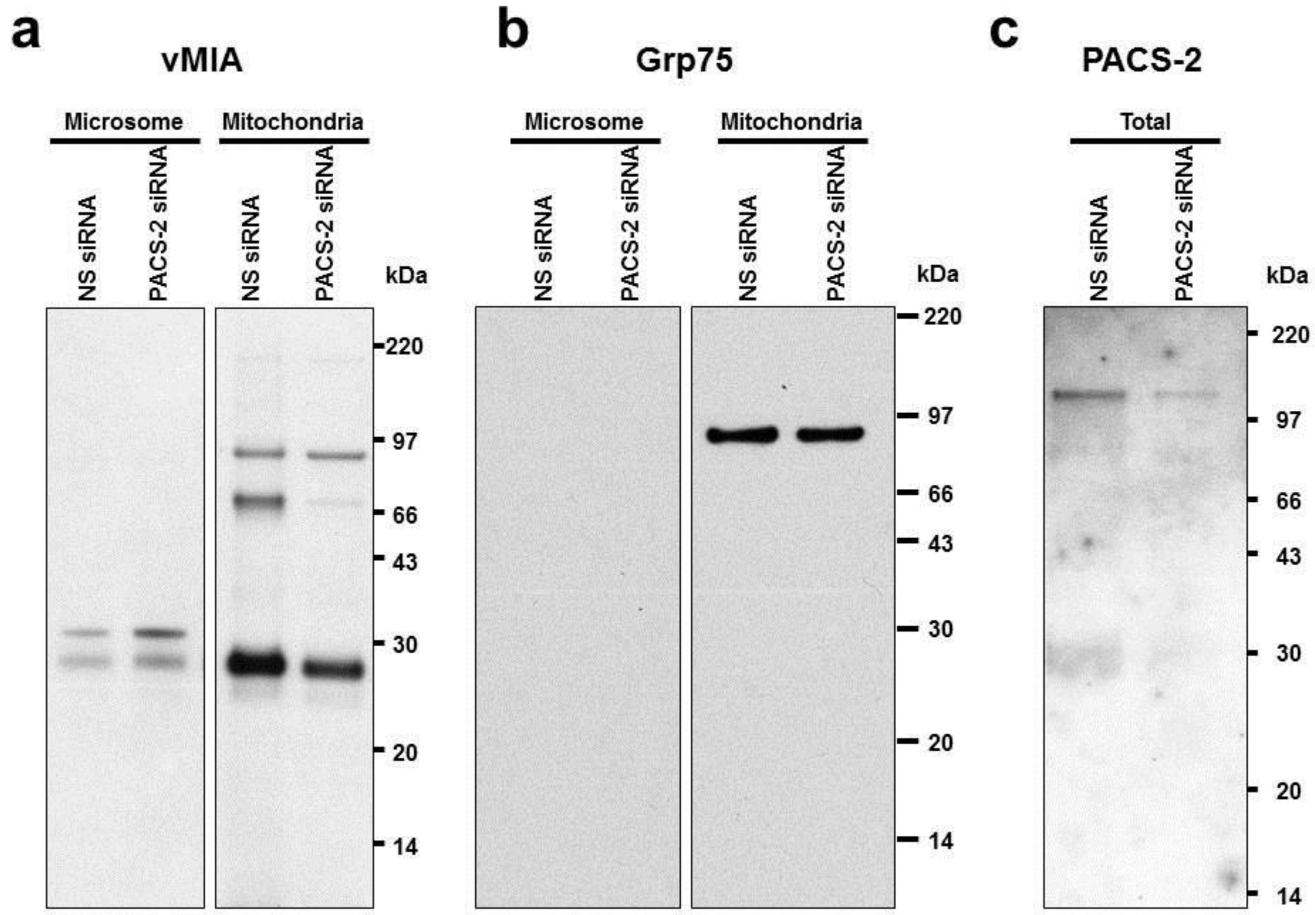


**Figure S5. Full Western blots for Figure 5h.** HeLa cells were lipofected with nonspecific siRNAs (NS siRNA) or PACS-2 siRNAs (PACS-2 siRNA) and with vector expressing WT vMIA and harvested 48 h after transfection. Microsomal and mitochondrial fractions were analyzed by Western blots as described in **Figure 5h**. Shown are the full-length blots for the Westerns used in **Figure 5h**: (**a**) vMIA and (**b**) Grp75 in microsomal and mitochondrial fractions and for (**c**) PACS-2 knockdown in total extracts from NS siRNA or PACS-2 siRNA treated transfected HeLa cells.
